# EIF2S1-PELO co-expression architecture in human brain: pairing-family decomposition reveals constitutive transcriptional coupling and functional layer separation

**DOI:** 10.3389/fnmol.2026.1817096

**Published:** 2026-08-10

**Authors:** Drake H. Harbert

**Affiliations:** Inner Architecture LLC, Canton, OH, United States

**Keywords:** weighted Jaccard, EIF2S1, PELO, ribosome rescue, co-expression network, pairing-family decomposition, Parkinson’s disease, layer separation

## Abstract

EIF2S1 (eIF2α, translation initiation surveillance) and PELO (Pelota, ribosome rescue) operate within established ribosome-associated quality control and translation-initiation surveillance pathways. Direct cross-talk between these systems is mediated through ZAK/GCN-mediated eIF2α phosphorylation under ribosome-stall stress. At the transcriptional layer, however, EIF2S1-PELO co-regulation has not been quantitatively characterized. Using pairing-family architectural decomposition on multi-region GTEx co-expression and BA9 prefrontal cortex RNA-seq from Parkinson’s disease and control donors (GSE68719, *n* = 44 controls, *n* = 29 PD), we find: (1) EIF2S1 and PELO share near-identical genome-wide co-expression architecture (continuous Weighted Jaccard = 0.914) with substantial top-partner overlap (binary Jaccard at top 5% = 0.490), producing a Type 1 dissociation gap of 0.424, significantly smaller than 200 random gene-pair gaps (mean 0.644, *z* = −2.57, permutation *p* = 0.015), and the lowest gap in a 21-pair quality-control gene panel; (2) the relationship is approximately linear-monotonic (Type 6 Pearson-Spearman gap ≈ 0.004); (3) cellular functional co-dependency in DepMap CRISPR screens is essentially zero (Jaccard = 0.006, gene-effect Pearson *r* = −0.07), consistent with layer separation between regulatory architecture and cellular phenotype; (4) in an exploratory single-cohort comparison of PD versus control prefrontal cortex, the dissociation gap was larger in PD (0.536 versus 0.424 in control), but this between-group difference did not reach statistical significance under a group-label permutation test (*p* = 0.40; bootstrap 95% CI on the difference [−0.14, 0.29]), so we present it as a hypothesis for replication rather than an established effect. These findings characterize EIF2S1-PELO transcriptional co-regulation as a constitutive architectural feature distinct from the ZAK/GCN-mediated direct mechanism. The constitutive coupling replicated in independent Alzheimer’s disease and ALS frontal-cortex cohorts and across microarray and RNA-seq platforms; a disease-associated weakening of the coupling was directionally consistent across all three diseases but did not reach statistical significance. We note that co-expression patterns are consistent with shared upstream regulatory programs but do not by themselves establish direct co-regulation; the architectural findings reported here are correlational at the transcriptional layer.

## 1 Introduction

Translation initiation surveillance and ribosome rescue are functionally linked components of the cell’s translation-surveillance and ribosome-rescue machinery. Translation initiation is regulated through phosphorylation of the alpha subunit of eIF2 (encoded by EIF2S1) under cellular stress conditions, including amino acid starvation, viral infection, ER stress, and oxidative stress, triggering the integrated stress response (ISR). Ribosome rescue is initiated when ribosomes stall on aberrant mRNAs; Pelota (PELO) functions in collision-induced No-Go Decay (NGD) and Non-Stop Decay (NSD) of truncated transcripts. Direct mechanistic cross-talk between these systems is established through the ZAK kinase/GCN2 axis: ribosome stalling activates ZAK, which phosphorylates eIF2α to initiate ISR or apoptosis (Wu et al., 2020).

At the transcriptional regulatory layer—a distinct level from direct biochemical cross-talk—co-expression network analysis can reveal whether genes show coordinated expression across cellular states that is consistent with shared upstream regulatory programs. Co-expression patterns are consistent with co-regulation through shared chromatin context, transcription factors, or condition-coupled programs, although co-expression alone does not establish direct co-regulation. Such co-expression coupling is independent of, and complementary to, direct functional interaction; it can exist between functionally independent genes and can be absent between functionally related genes whose direct interaction is post-transcriptional or constitutive at protein level. Here, we apply pairing-family architectural decomposition (Harbert, 2026) to characterize EIF2S1-PELO transcriptional co-expression as a layer of biology distinct from established direct mechanisms.

The pairing-family decomposition treats two related similarity metrics, applied to the same data, as paired measurements. The continuous Weighted Jaccard (WJ) on the full coupling profile and the binary Jaccard at top-percentile partners are continuous and discrete versions of the same underlying mathematical object: binary Jaccard is the thresholded limit of WJ. The gap between them—the Type 1 dissociation gap—localizes where in the value distribution architectural divergence concentrates (broad-distribution layer vs. top-partner layer). This decomposition was validated on SIGMAR1-TMEM97 in Harbert (2026) and is applied here to the EIF2S1-PELO pair and a 21-pair quality-control gene panel.

Three additional measurements complement the Type 1 analysis: Type 2 (sign-treatment family) characterizes whether the architectural similarity is sign-driven; Type 6 (substrate-projection) compares Spearman versus Pearson realizations to characterize linear-monotonic structure; and DepMap CRISPR co-dependency analysis tests whether the transcriptional coupling translates to functional cellular interdependence. Disease-state modulation of the architecture is assessed in BA9 prefrontal cortex from Parkinson’s disease donors (Dumitriu et al., 2016), a neurodegenerative disease in which translational dysregulation has been implicated.

## 2 Methods

### 2.1 Data sources

Multi-region brain co-expression analysis used Genotype-Tissue Expression Project (GTEx) v8 RNA-seq for five brain regions: Brodmann Area 9 (BA9, frontal cortex), basal ganglia (caudate, putamen, nucleus accumbens), Brodmann Area 24 (BA24, anterior cingulate), and hippocampus. Disease-state comparison used GSE68719 (Dumitriu et al., 2016): BA9 prefrontal cortex RNA-seq from 29 Parkinson’s disease donors and 44 control donors, DESeq2-normalized counts. Functional co-dependency analysis used DepMap (Tsherniak et al., 2017) CRISPR gene-effect data, release 24Q2, comprising knockout phenotypes across 1,150 cancer cell lines.

Two additional neurodegenerative-disease cohorts were used as independent replication of the EIF2S1-PELO coupling. Alzheimer’s disease: GSE5281 (Liang et al., 2008), superior frontal gyrus, 23 Alzheimer’s and 11 control samples, Affymetrix HG-U133 Plus 2.0 microarray, an independent platform from the RNA-seq primary and PD analyses. Amyotrophic lateral sclerosis: GSE124439 (Tam et al., 2019), frontal cortex, 65 ALS Spectrum MND and 9 non-neurological control samples, RNA-seq (New York Genome Center ALS Consortium). For the microarray cohort, probe values were collapsed to genes by mean and used directly, since Spearman correlation is rank-based and scale-invariant; for the ALS cohort, STAR gene counts were normalized to log2 counts-per-million with an expressed-gene filter of median CPM at least 1. Both cohorts were analyzed with the pipeline used for the PD comparison (Sections 2.2 and 2.3); a stratified-permutation meta-test (5,000 permutations) combined the two RNA-seq cohorts (PD, ALS) in the PD-established direction.

### 2.2 Coupling profile and pairing-family decomposition

For each gene g and a sample set S, the coupling profile c_g is the vector of gene-level Spearman rank correlations between g and every other expressed gene (median DESeq2-normalized count ≥5). Pairing-family decompositions were computed on the EIF2S1 and PELO coupling profiles as follows.

*Type 1 (Continuous-Discrete dissociation gap)*: Continuous Weighted Jaccard on shifted values (*r* + 1)/2: WJ_shifted (c_X, c_Y) = Σ min((c_X + 1)/2, (c_Y + 1)/2)/Σ max((c_X + 1)/2, (c_Y + 1)/2). Binary Jaccard at top 5%: J_5 (c_X, c_Y) = |TopP_X ∩ TopP_Y|/|TopP_X ∪ TopP_Y|, where TopP is the gene set in the top 5% of |*r*| values for the corresponding coupling profile. Dissociation gap = WJ_shifted − J_5.

*Type 2 (Sign-treatment family)*: Three formulations on the same coupling profiles: WJ_shifted (above), WJ_unsigned on |c|, and WJ_signed computed by splitting positive and negative components separately. Sign-treatment gaps quantify the contribution of correlation sign to architectural similarity.

*Type 6 (Substrate-projection)*: Spearman-derived coupling profiles vs. Pearson-derived coupling profiles, with continuous and binary metrics computed on each. Type 6 gap = Spearman_WJ − Pearson_WJ. Near-zero gap indicates approximately linear-monotonic relationship structure.

### 2.3 Statistical testing

*Permutation null*: To assess whether the EIF2S1-PELO Type 1 dissociation gap is non-random, 200 random gene-pair gaps were computed using the same metrics on the same control sample set. Two-sided permutation *p*-value reports the proportion of |null gaps − null mean| ≥ |observed − null mean|. To test whether the dissociation gap differs between PD and control, a separate between-group permutation test relabeled the pooled PD and control samples at random (2,000 relabelings), recomputing each pseudo-group’s gap and the difference between the PD and control gaps; a subject-level bootstrap (1,000 iterations) provided a 95% confidence interval on that difference.

*Bootstrap confidence interval*: The EIF2S1-PELO Type 1 gap was bootstrapped by resampling subjects with replacement (500 iterations); the 2.5–97.5% percentile interval is reported as the 95% bootstrap CI.

*21-pair landscape and Fisher OR*: Pairwise comparisons were computed for the seven-gene quality-control panel (EIF2S1, PELO, LTN1, NEMF, TMEM97, HSPA5, SIGMAR1)—twenty-one pairs in total. Fisher exact odds ratios (OR) for top-5% partner overlap quantify biological relatedness independent of the WJ-derived gap.

Reproducibility. RANDOM_SEED = 42 throughout. FORCE_RECOMPUTE = True. All analyses are reproducible from a single-command Python pipeline; see Section 4 (Data and Code Availability).

## 3 Results

### 3.1 Type 1 dissociation gap is the lowest in a 21-pair quality-control panel and is significantly smaller than random gene-pair gaps

**Figure 1:**
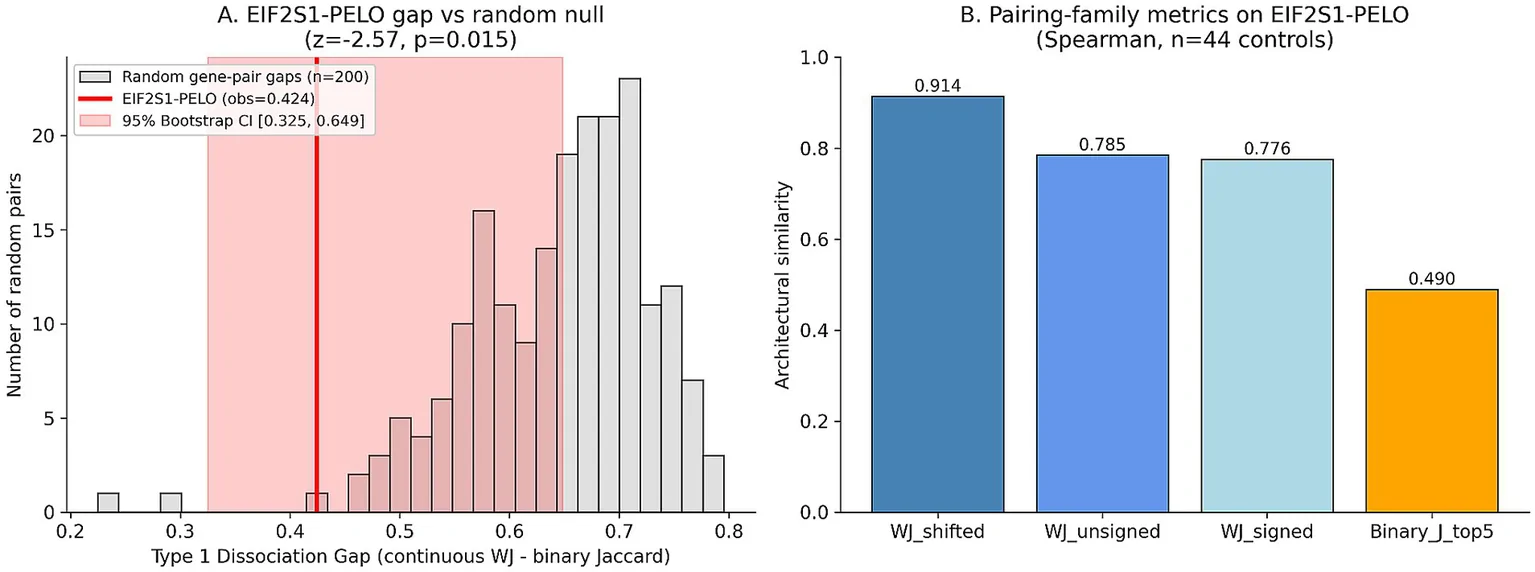
Pairing-family decomposition of the EIF2S1-PELO coupling profile and its permutation null in BA9 control cortex. **(A)** The EIF2S1-PELO Type 1 dissociation gap (continuous WJ_shifted minus binary Jaccard at the top 5% threshold) shown against the distribution of 200 random gene-pair gaps computed under the same protocol; the observed gap lies 2.57 standard deviations below the random-pair mean (permutation *p* = 0.015, two-sided). **(B)** Pairing-family metrics computed on the EIF2S1-PELO coupling profiles in the GSE68719 control samples (*n* = 44): continuous WJ_shifted = 0.914, binary Jaccard at top 5% = 0.490, and Type 1 dissociation gap = 0.424 (bootstrap 95% CI 0.325 to 0.649).

**Figure 2:**
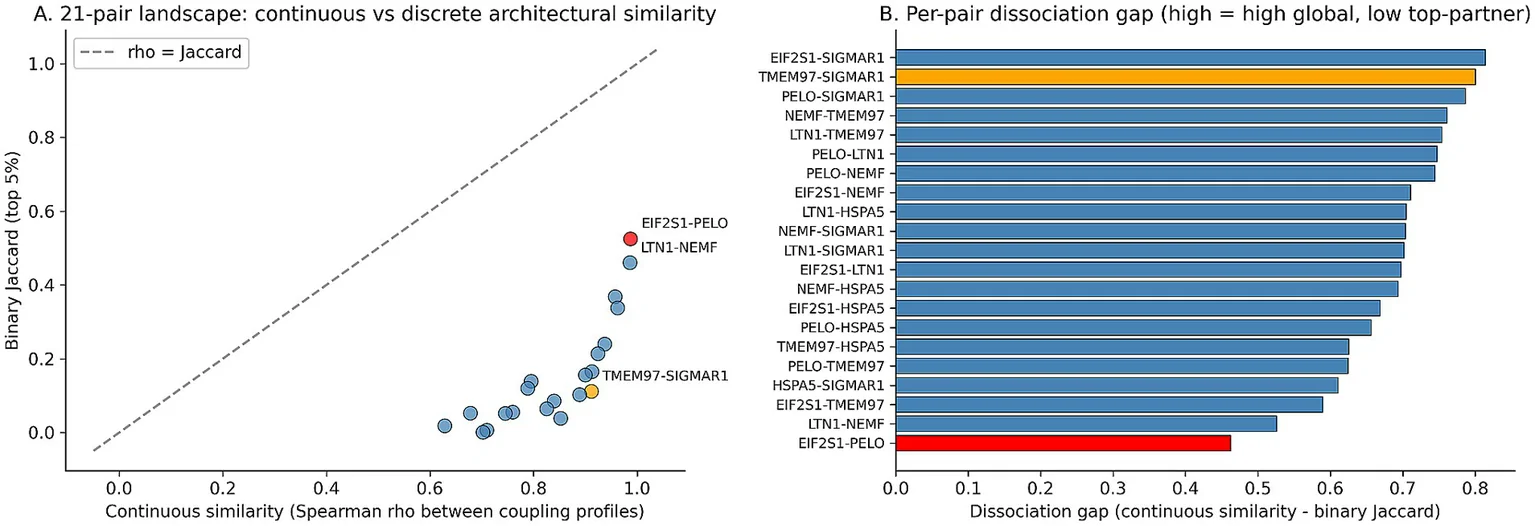
Twenty-one-pair quality-control panel landscape. Type 1 dissociation gaps for all pairwise comparisons among the seven-gene quality-control panel (EIF2S1, PELO, LTN1, NEMF, TMEM97, HSPA5, SIGMAR1), ranging from 0.46 (EIF2S1-PELO, the lowest and closest-coupled) to 0.81 (EIF2S1-SIGMAR1, the highest). EIF2S1-PELO occupies the closest-coupled position in the panel and shows the highest Fisher odds ratio for shared top partners (132.39). **(A)** Scatter of continuous coupling-profile similarity (Spearman rho between coupling profiles) versus binary Jaccard at the top 5% threshold for all 21 gene pairs, with the equality line shown. **(B)** The 21 gene pairs ranked by Type 1 dissociation gap (continuous WJ shifted minus binary Jaccard), from the lowest, closest-coupled extreme (EIF2S1-PELO) to the highest.

Pairing-family decomposition of EIF2S1-PELO coupling profiles in BA9 control samples (*n* = 44, GSE68719) yielded continuous WJ_shifted = 0.914 and binary Jaccard at top 5% = 0.490, producing a Type 1 dissociation gap of 0.424 (bootstrap 95% CI: 0.325 to 0.649; Table 1, Figure 1). The same decomposition applied to a 21-pair quality-control panel (Figure 2), computed on the multi-region GTEx co-expression data rather than the GSE68719 BA9 control set, produced gaps ranging from 0.46 (EIF2S1-PELO, the lowest and hence closest-coupled) to 0.81 (EIF2S1-SIGMAR1, the highest); the small difference from the BA9 value above reflects the different dataset. EIF2S1-PELO occupied the closest-coupled position in the panel, with the highest Fisher OR for shared partners (132.39).

**Table 1:** EIF2S1-PELO Type 1 dissociation gap with statistical anchoring (BA9 prefrontal cortex, GSE68719 controls, *n* = 44).

Measurement	Value
Continuous WJ (shifted, (*r* + 1)/2)	0.9142
Continuous WJ (unsigned, |*r*|)	0.7851
Continuous WJ (signed split)	0.7759
Binary Jaccard at top 5%	0.4898
Type 1 dissociation gap (shifted − binary)	0.4245
Bootstrap mean (500 iterations)	0.4879
Bootstrap 95% CI lower	0.3252
Bootstrap 95% CI upper	0.6485
Permutation null mean (200 random pairs)	0.644
Permutation *z*-score	−2.57
Permutation *p*-value (two-sided)	0.0150
*N* samples	44
*N* genes	16,010

Permutation null computed against 200 random gene pairs from the same expression matrix using identical metrics. The EIF2S1-PELO gap is significantly smaller than random expectation, indicating non-random architectural agreement between continuous and discrete realizations of the coupling-profile similarity.

To assess whether the EIF2S1-PELO dissociation gap is non-random, 200 random gene-pair gaps were computed under the same protocol. Random pairs produced a mean gap of 0.644 (SD 0.086), indicating that random gene pairs typically show much weaker continuous-discrete agreement than EIF2S1-PELO. The observed EIF2S1-PELO gap was 2.57 SD below the random-pair mean (permutation *p* = 0.015, two-sided). Random gene pairs cluster at higher gaps because their continuous similarity is moderate while their top-partner overlap is low; EIF2S1-PELO show comparatively tight agreement between continuous and discrete layers, consistent with biologically coupled co-expression that may reflect shared upstream regulation.

### 3.2 Architecture is approximately linear-monotonic and moderately sign-driven

Type 6 substrate-projection analysis (Spearman-derived versus Pearson-derived coupling profiles) yielded a near-zero gap on the continuous metric (0.004; Spearman WJ = 0.914 vs. Pearson WJ = 0.910) and a small binary gap (−0.05). Per the principled-pairing criterion (Harbert, 2026), the near-null Type 6 gap is itself an informative measurement: it indicates that EIF2S1-PELO coupling structure is approximately linear-monotonic in this substrate, independent of monotonic transformation. This simplifies downstream interpretation and rules out nonlinear-only relationships.

Type 2 sign-treatment analysis revealed moderate sign contribution. The WJ_shifted, WJ_unsigned, and WJ_signed formulations produced values of 0.914, 0.785, and 0.776 respectively, with maximum pairwise gap of 0.138 between shifted and signed formulations. This indicates that correlation sign carries non-trivial information in the EIF2S1-PELO coupling architecture—neither dominant nor negligible. Following the framework’s pairing-family principles, both signed and unsigned WJ are reported transparently; the moderate sign contribution suggests interpretation should not rely solely on either signed or unsigned formulations alone.

### 3.3 EIF2S1-PELO co-expression architecture is constitutive across human brain regions

**Figure 3:**
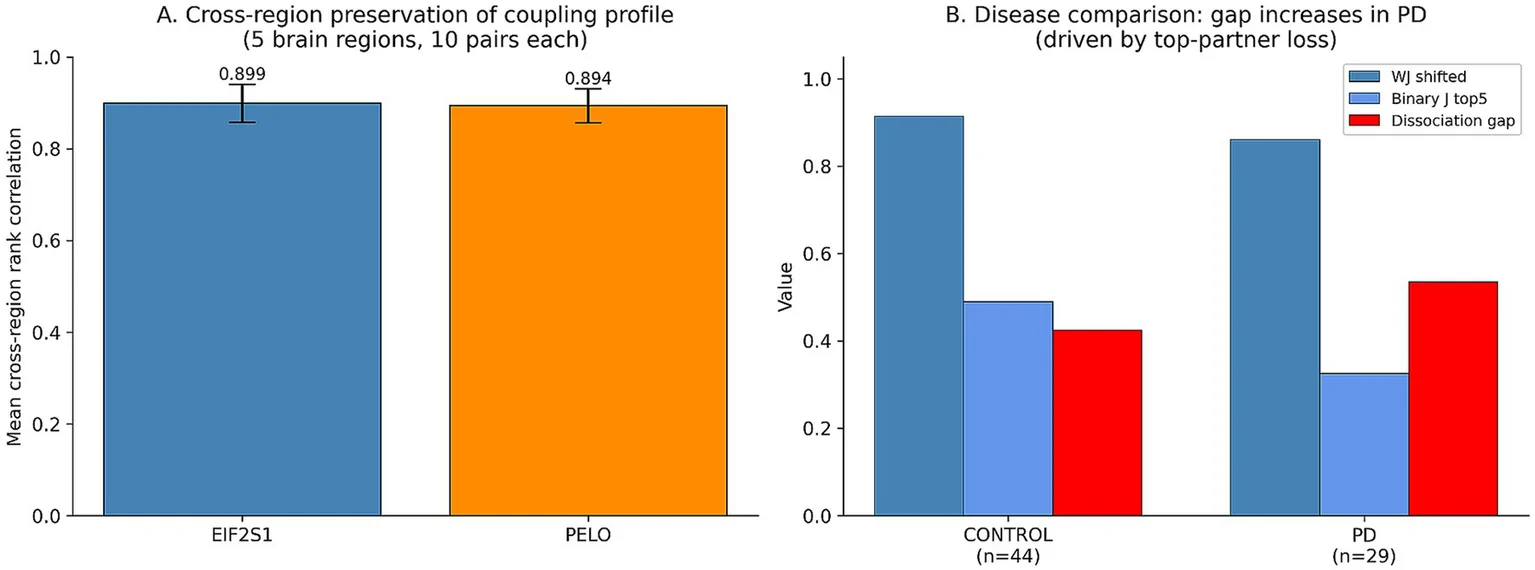
Cross-region preservation of the EIF2S1-PELO architecture and an exploratory Parkinson’s disease comparison. **(A)** Cross-region preservation of the EIF2S1 and PELO coupling profiles across five human brain regions (GTEx v8): Mean rank correlations 0.899 (EIF2S1) and 0.894 (PELO), not significantly different (Mann–Whitney *p* = 0.47). **(B)** Exploratory comparison in GSE68719 BA9 prefrontal cortex: The EIF2S1-PELO Type 1 dissociation gap was larger in PD (0.536, *n* = 29) than in control (0.424, *n* = 44), with continuous WJ_shifted 0.914 to 0.861 and binary Jaccard at top 5% 0.490 to 0.326. This between-group difference did not reach statistical significance (permutation *p* = 0.40; bootstrap 95% CI [−0.14, 0.29]) and is shown as a hypothesis-generating trend, not an established effect.

Multi-region GTEx analysis (Figure 3A) identified EIF2S1 and PELO coupling profiles preserved across the five brain regions tested with mean rank correlations of 0.899 (EIF2S1) and 0.894 (PELO). The two genes did not differ significantly in cross-region preservation (Mann–Whitney *p* = 0.47), consistent with both genes being subject to the same cross-region regulatory program.

### 3.4 Layer separation: zero functional cellular co-dependency in DepMap CRISPR screens

**Figure 4:**
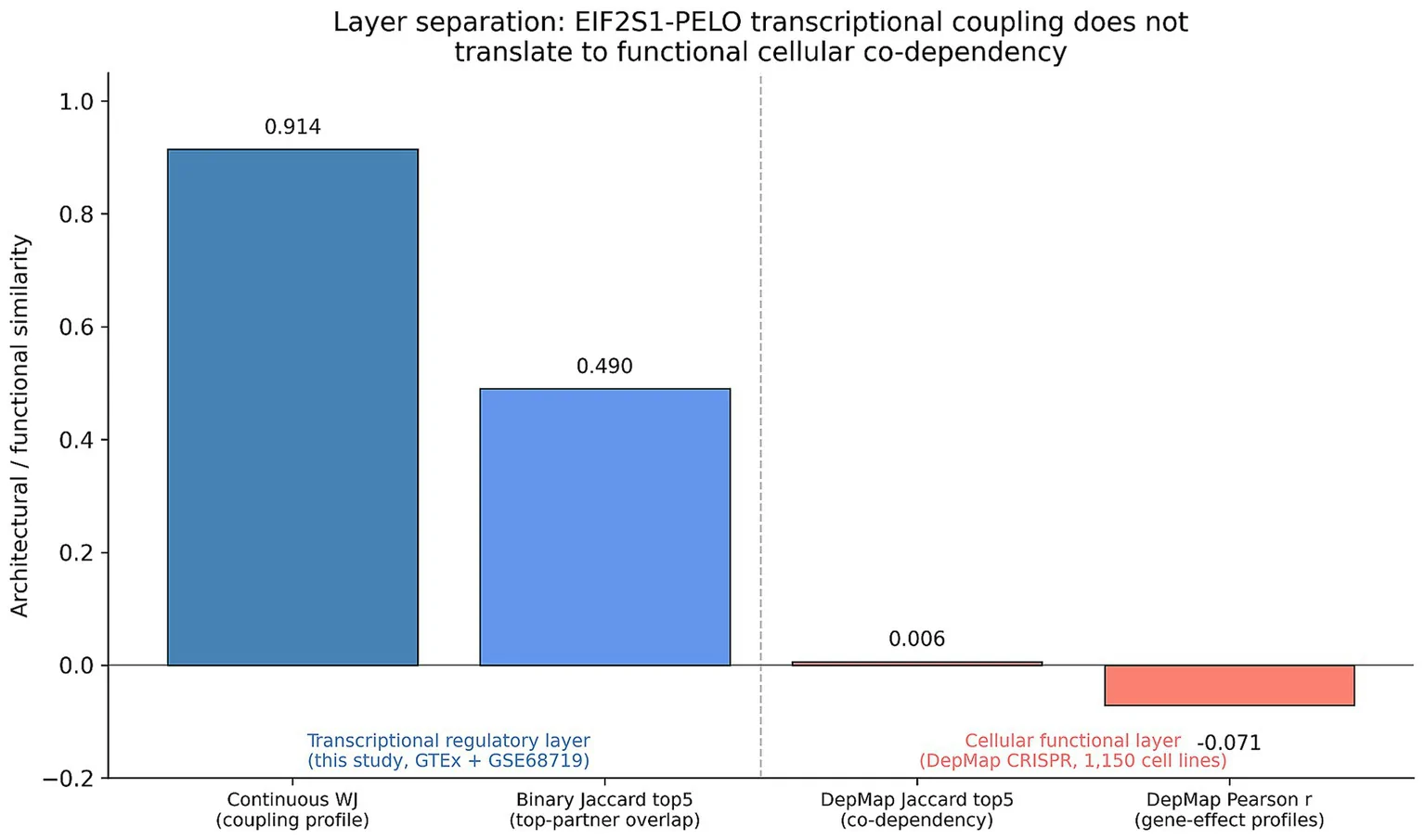
Layer separation between transcriptional coupling and functional cellular co-dependency: Top 5% Jaccard = 0.006 (11 shared dependencies of 890 each), Fisher exact *p* = 0.9999 (under-enrichment direction), and Pearson correlation between the gene-effect profiles = −0.07.

DepMap (Tsherniak et al., 2017) gene-effect data were used to test whether EIF2S1-PELO transcriptional coupling translates to functional co-dependency in cellular phenotypes. Across 1,150 cancer cell lines, the EIF2S1 and PELO gene-effect profiles showed essentially zero co-dependency: top 5% Jaccard of 0.006 (11 shared dependencies of 890 each), Fisher exact *p* = 0.9999 (under-enrichment direction), and direct Pearson correlation between gene-effect profiles of −0.07 (Figure 4, Table 2). EIF2S1 and PELO are tightly transcriptionally co-regulated in human brain (Section 3.1) but functionally independent in cellular phenotypic screens.

**Table 2:** Layer separation between transcriptional regulatory architecture and cellular functional co-dependency for EIF2S1-PELO.

Layer	Metric	Value	Interpretation
Co-expression coupling architecture (this manuscript)	Spearman rho between coupling profiles	0.9873	Near-identical genome-wide coupling architecture
Top-partner overlap (this manuscript)	Binary Jaccard at top 5% threshold	0.5249	Modest overlap at strongest correlation partners
Functional co-dependency (DepMap CRISPR)	Co-dependency Jaccard at top 5%	0.0062	Essentially zero functional cellular co-dependency
Functional co-dependency (DepMap CRISPR)	Pearson r of gene-effect profiles	−0.0714	No correlation in CRISPR phenotypic effects across cell lines

Transcriptional regulatory layer derived from GSE68719 BA9 controls (*n* = 44). Cellular functional layer derived from DepMap (Tsherniak et al., 2017) CRISPR gene-effect data, release 24Q2, across 1,150 cancer cell lines.

An independent protein-interaction analysis using the STRING database (v12, *Homo sapiens*) corroborates this layer separation at the level of physical interactions (Supplementary Figure S1). The STRING panel comprised 10 genes: six of the seven-gene co-expression panel (EIF2S1, PELO, LTN1, NEMF, TMEM97, SIGMAR1) together with four ribosome-quality-control extension genes examined in this study (TCF25, ASCC1, ASCC3, TRIP4); HSPA5 was not included in the STRING panel. Among these 10 genes, STRING reports no direct EIF2S1-PELO interaction at medium confidence, while the panel as a whole is strongly interaction-enriched (14 edges observed versus approximately zero expected; PPI enrichment *p* = 1.1*e*−16). The top STRING interaction partners of the two genes occupy separate functional layers: EIF2S1 with translation-initiation factors and the eIF2 kinases EIF2AK2 and EIF2AK4, and PELO with the ribosome-rescue factors HBS1L, ABCE1, and RACK1. The genes are therefore embedded in a coherent interaction module yet are not direct binding partners, consistent with transcriptional co-regulation rather than a direct protein complex.

This dissociation between transcriptional coupling and functional co-dependency is consistent with layer separation in the regulatory biology linking translation-initiation surveillance and ribosome-associated quality control. The transcriptional coupling is consistent with shared upstream regulatory programs (chromatin context, common transcription factors, condition-coupled stress-response programs); the functional independence indicates that cellular phenotypic effects of either gene’s perturbation do not require the other. The two layers carry complementary biological information and provide a more complete picture than either alone.

### 3.5 Exploratory comparison of top-partner architecture in Parkinson’s disease and control BA9 prefrontal cortex

Stratifying GSE68719 into PD (*n* = 29) and control (*n* = 44) subsets, the EIF2S1-PELO Type 1 dissociation gap was larger in PD (0.536) than in control (0.424) (Figure 3B, Table 3). Continuous WJ_shifted decreased modestly (0.914 in control to 0.861 in PD), while binary Jaccard at top 5% decreased more (0.490 to 0.326). We tested this between-group difference directly by relabeling the pooled PD and control samples at random (2,000 permutations). The observed gap difference (0.111) did not reach significance (permutation *p* = 0.40), and a subject-level bootstrap 95% confidence interval on the difference ([−0.14, 0.29]) included zero; the top-partner overlap drop was likewise non-significant (permutation *p* = 0.35). We therefore present the PD comparison as an exploratory, hypothesis-generating observation in a single cohort rather than an established disease effect. The direction is consistent with preferential loss of the strongest-coupled partner layer, but at this sample size the difference is not statistically distinguishable from sampling variation.

**Table 3:** EIF2S1-PELO architectural metrics in Parkinson’s disease and control BA9 prefrontal cortex (GSE68719).

Cohort	*N* samples	Continuous WJ (shifted)	Binary Jaccard top 5%	Type 1 dissociation gap
Control	44	0.9142	0.4898	0.4245
PD	29	0.8612	0.3256	0.5356

PD vs. control comparison shows that the dissociation gap increases in PD (0.424 → 0.536), driven primarily by selective loss of binary top-partner overlap (0.490 → 0.326) while continuous architectural similarity is largely preserved (0.914 → 0.861). This identifies the top-partner architectural layer as a candidate site of disease-selective vulnerability.

This direction is consistent with PD-associated dysregulation of the initiation-surveillance and ribosome-rescue programs, in which high-confidence top-partner co-expression would erode while the broader regulatory context remains partially intact. Together with the published association of translational dysregulation with α-synuclein pathology and PD progression (Hetz and Saxena, 2017; Dumitriu et al., 2016), this exploratory pattern nominates the top-partner architectural layer as a candidate site for disease-associated vulnerability, to be tested in future adequately powered cohorts.

### 3.6 Replication of the constitutive coupling across additional diseases and an independent platform

To test whether the EIF2S1-PELO coupling generalizes beyond GTEx and the PD cohort, the identical pairing-family analysis was applied to Alzheimer’s disease superior frontal gyrus (GSE5281) and amyotrophic lateral sclerosis frontal cortex (GSE124439). The constitutive coupling replicated in both: WJ_shifted in control tissue was 0.822 (AD) and 0.850 (ALS), consistent with GTEx and the PD control set (0.914) and robust to the RNA-seq versus microarray platform difference. In disease, the coupling weakened in all three diseases (WJ_shifted change of −0.053 in PD, −0.112 in AD, and −0.098 in ALS), and the Type 1 dissociation gap increased in both RNA-seq cohorts (PD + 0.111, ALS + 0.199). None of the between-group changes reached significance individually (permutation *p* = 0.40 for PD, 0.27 for AD, 0.10 for ALS), and a stratified-permutation meta-test combining the two RNA-seq cohorts in the PD-established direction gave *p* = 0.10 (Supplementary Table S13). The disease-associated change is therefore directionally consistent across three independent neurodegenerative diseases but remains statistically unconfirmed at present sample sizes, and we interpret it as a hypothesis-generating trend rather than an established effect. Because the binary top-partner overlap was near zero on the microarray platform, the AD dissociation gap is not directly comparable to the RNA-seq cohorts, and the AD cohort is interpreted on the continuous coupling metric only. The replication of the constitutive coupling across four cohorts, three diseases, and two platforms is the primary result of this analysis and supports the manuscript’s constitutive-coupling conclusion independent of any disease-associated change.

## 4 Discussion

We have shown that EIF2S1 and PELO, translation initiation surveillance and ribosome rescue components respectively, share constitutive transcriptional co-expression in human brain at near-perfect global fidelity, with substantial top-partner overlap that significantly exceeds random expectation, and with a dissociation-gap signature representing the closest-coupled position in a 21-pair quality-control panel. This transcriptional coupling is independent of cellular functional co-dependency (DepMap) and occurs at a distinct biological layer from the established direct ZAK/GCN-mediated cross-talk (Wu et al., 2020). In an exploratory single-cohort comparison, the PD BA9 prefrontal cortex showed a direction consistent with preferential loss of the top-partner layer, though this between-group difference did not reach statistical significance and requires replication.

The framework distinction between regulatory architecture and cellular function clarifies what gene co-expression analysis can and cannot infer. High continuous WJ combined with substantial binary Jaccard is consistent with two genes being co-regulated through shared upstream programs across cellular states, although co-expression alone does not establish direct co-regulation; alternative explanations include shared cell-type composition, coincident expression in similar cellular states, and downstream coordination through common regulators. Co-expression also does not establish that the genes are functionally interdependent. The DepMap result here makes this last distinction empirical: EIF2S1 and PELO are co-regulated at the transcriptional layer but not at the functional layer. This architecture is consistent with the established mechanistic biology (Wu et al., 2020): direct cross-talk through ZAK/GCN-mediated eIF2α phosphorylation occurs in the rapid stress-response biochemical pathway, while baseline transcriptional co-expression maintains coordinated expression of both modules under non-stressed conditions. The two pathways are complementary, not redundant; one operates at the post-transcriptional biochemical level, the other at the regulatory architecture level.

The exploratory PD comparison provides an architectural hypothesis for translational-surveillance and ribosome-rescue dysregulation in neurodegeneration, though the between-group difference did not reach statistical significance in this single cohort and requires replication. Rather than a wholesale collapse of EIF2S1-PELO co-expression, what appears to change in PD is the strongest-coupled layer of the architecture: pairs that are most tightly correlated with both genes in the healthy state lose that consensus in disease. The broad regulatory context, the genome-wide pattern of who is co-expressed with whom in general, is largely preserved. This pattern suggests that PD-associated translational dysregulation may selectively erode the highest-confidence co-expression relationships, possibly through partial decoupling of specific stress-response or housekeeping programs that under healthy conditions anchor both EIF2S1 and PELO to a shared partner set.

### 4.1 Methodological note: pairing-family decomposition

Reviewers of co-expression network analyses sometimes raise concerns about absolute Jaccard magnitudes—for instance, that maximum values below 1.0 imply weak coupling. This concern misreads what binary Jaccard measures. The Jaccard scale is bounded but its absolute value depends on substrate connectivity density and threshold choice. The principled-pairing criterion (Harbert, 2026) reframes the question: rather than asking whether absolute Jaccard is high enough, the framework asks how the continuous and discrete realizations of the same underlying metric agree with each other. The Type 1 dissociation gap localizes architectural divergence by its position in the value distribution; the gap’s distance from random-pair-null distributions provides a basis for evaluating statistical significance of the observed agreement.

For EIF2S1-PELO, this framework provides two independent statistical anchors: (1) the gap is significantly smaller than 200 random-pair null gaps (*z* = −2.57, permutation *p* = 0.015); and (2) the gap is the lowest in a 21-pair quality-control panel, indicating EIF2S1-PELO occupy the closest-coupled extreme of the local architectural landscape. Absolute Jaccard alone would not have produced these inferences; the pairing-family framework extracts them by treating the continuous and discrete metrics as paired measurements.

Terminology note: ribosome rescue mechanisms are themselves heterogeneous, encompassing No-Go Decay (NGD), Non-Stop Decay (NSD), and Ribosome Quality Control Trigger (RQT) and Ribosome-associated Quality Control (RQC) pathways. PELO functions specifically in NGD and NSD; the analyses here characterize co-expression relationships within and across these pathways but do not adjudicate which specific sub-process is most tightly coupled to EIF2S1.

### 4.2 Limitations

Findings are correlational at the transcriptional layer; direct interaction between EIF2S1 and PELO proteins, as well as the upstream regulatory programs driving their co-expression, were not addressed in this analysis. The DepMap analysis used cancer cell lines and may not fully reflect functional dependencies in brain tissue or neurodegenerative states; brain-specific functional co-dependency screens would strengthen the layer-separation interpretation. The PD analysis was conducted in a single cohort (GSE68719); replication in additional PD or other neurodegenerative cohorts is the natural next step. The 21-pair panel was restricted to a focused quality-control gene set; broader landscape analyses across thousands of pairs would help characterize the population distribution of dissociation gaps for biologically relevant pairs.

## Data Availability

Publicly available datasets were analyzed in this study. GTEx v8 (https://gtexportal.org/, dbGaP phs000424.v8.p2); GSE68719, GSE5281, and GSE124439 (NCBI GEO, https://www.ncbi.nlm.nih.gov/geo/); and DepMap release 24Q2 (https://depmap.org/). All analysis code is openly available at https://github.com/nwharbert8-ui/eif2s1-translational-qc-hubsub and archived at Zenodo (https://doi.org/10.5281/zenodo.18626362).

## Supplementary material

SUPPLEMENTARY FIGURE S1 STRING protein-protein interaction network for the ten-gene panel (STRING v12, Homo sapiens). No direct EIF2S1-PELO interaction is reported at medium confidence, while the panel as a whole is strongly interaction-enriched (14 edges observed versus approximately zero expected; PPI enrichment p = 1.1e-16). The top interaction partners of the two genes occupy separate functional layers: EIF2S1 with translation-initiation factors and the eIF2 kinases EIF2AK2 and EIF2AK4, and PELO with the ribosome-rescue factors HBS1L, ABCE1, and RACK1.
