# Supplementary material for: EIF2S1-PELO co-expression architecture in human brain: pairing-family decomposition reveals constitutive transcriptional coupling and functional layer separation
Source: Front Mol Neurosci. 2026 Aug 10;19:1817096. doi: 10.3389/fnmol.2026.1817096 (PMC13500769; doi:10.3389/fnmol.2026.1817096)
Supplement: Supplementary file 1 [file Data_Sheet_13.docx]

**Supplementary Table S13. EIF2S1-PELO coupling and dissociation gap across disease cohorts.**

| **Cohort** | **Disease** | **Region** | **Platform** | **n ctrl** | **n dis** | **ctrl WJ** | **dis WJ** | **ctrl gap** | **dis gap** | **gap diff** | **perm p** |
| --- | --- | --- | --- | --- | --- | --- | --- | --- | --- | --- | --- |
| GSE68719 | PD | BA9 | RNA-seq | 44 | 29 | 0.914 | 0.861 | 0.424 | 0.536 | +0.111 | 0.40 |
| GSE5281 | AD | sup. frontal gyrus | microarray | 11 | 23 | 0.822 | 0.710 | 0.822 | 0.706 | -0.116 | 0.27 |
| GSE124439 | ALS | frontal cortex | RNA-seq | 9 | 65 | 0.850 | 0.752 | 0.543 | 0.742 | +0.199 | 0.10 |
| Meta (PD+ALS) | - | - | RNA-seq | - | - | - | - | - | - | +0.310 sum | 0.10 |

*WJ = WJ_shifted continuous coupling; gap = Type 1 dissociation gap (WJ_shifted minus binary Jaccard at top 5%); perm p = two-sided label-permutation p on the between-group gap difference. The AD gap is not directly comparable across the microarray/RNA-seq platform boundary (near-zero top-partner overlap on microarray); AD is interpreted on the WJ coupling metric. Meta = stratified-permutation test combining the two RNA-seq cohorts in the PD-established direction (one-sided). seed=42.*
